# Do Lactating Mothers’ Descriptions of Breastfeeding Pain Align with a Biopsychosocial Pain Reasoning Tool? A Qualitative Study

**DOI:** 10.3390/brainsci15101087

**Published:** 2025-10-08

**Authors:** Lester E. Jones, Lisa H. Amir, Nicole Shi En Chew, Shi Yun Low, Victoria Yu Ting Woo, Doris Fok, Yvonne Peng Mei Ng, Zubair Amin

**Affiliations:** 1 Health and Social Sciences, Singapore Institute of Technology, Singapore 828608, Singapore; lester.jones@singaporetech.edu.sg (L.E.J.);; 2 Judith Lumley Centre, School of Nursing and Midwifery, La Trobe University, Bundoora 3086, Australia; 3 Breastfeeding Service, Royal Women’s Hospital, Parkville 3052, Australia; 4 Department of Neonatology, Khoo Teck Puat—National University Children’s Medical Institute, National University Hospital, Singapore 119228, Singaporepaeza@nus.edu.sg (Z.A.); 5 Department of Paediatrics, National University of Singapore, Singapore 117597, Singapore

**Keywords:** breastfeeding, clinical reasoning, lactation, biopsychosocial, pain, interoception, women’s health

## Abstract

**Background/Objectives:** Despite the intent of most mothers to breastfeed their children, breast or nipple pain can be the reason for early cessation of breastfeeding. Current understanding about lactation-related pain revolves around mechanical or pathological causes, discounting the role of psychosocial factors which can influence management of pain. The Breastfeeding Pain Reasoning Model is a clinical reasoning tool developed to support those evaluating women’s lactation-related pain. We aimed to explore perspectives of breastfeeding women on lactation-associated pain and determine how they align with the Breastfeeding Pain Reasoning Model (BPRM). **Methods:** We conducted a qualitative descriptive study using phenomenological approach in Singapore. Eighteen women with recent breast and/or nipple pain during lactation underwent individual semi-structured interviews in 2022. Thematic analysis was performed. **Results:** Deductive analysis showed that lactation-associated pain was aligned with BPRM’s domains (i.e., local stimulation, external influences, and central modulation). Psychosocial factors likely influencing central processing of pain were not recognised by most of the participants. Participants described severe breastfeeding pain often accompanied by feelings of vulnerability, injustice, and uncertainty. Inductive analysis identified two additional themes of motivation and expectations. **Conclusions:** Greater awareness of the interplay between the broad influences on pain is needed. Using an interoceptive lens could help to illustrate how signals from the breast inform the brain, and how social, emotional, and cognitive factors influence the individuals’ perception of painful experiences. Educating breastfeeding women and healthcare personnel about the biopsychosocial nature of pain may empower women to better navigate the challenges of breastfeeding and improve breastfeeding outcomes.

## 1. Introduction

Although most mothers plan to breastfeed their children [1,2], many stop breastfeeding earlier than intended and do not meet the World Health Organization’s recommendation for six months of exclusive breastfeeding followed by continued breastfeeding for two years or beyond [3,4,5]. Concerns about insufficient milk production are common and many mothers receive little education about how to attach the baby to the breast or establish successful breastfeeding [6]. One large study in the USA found that less than half of women breastfeed as long as they intended [7]. About 60% reported sore nipples/breast tenderness [7], and breast or nipple pain is commonly implicated in reasons for early cessation of breastfeeding [8,9].

In addition to stopping breastfeeding prematurely, nipple/breast pain is distressing for new mothers who are recovering from childbirth and adapting to life as a new mother [10]. Breastfeeding pain may increase their risk of anxiety and depression [11,12,13]. Many mothers report being unprepared for breastfeeding and experience long-term guilt and disappointment if they experienced challenges with breastfeeding [14].

Credible models and evidence of the complex nature of pain have been available for many years [15,16,17,18,19]. Despite this, existing research on lactation-related pain has focussed on mechanical or pathological causes [20,21]. Stress, fatigue, and inadequate sleep are often experienced by breastfeeding mothers [7] and, along with anxiety, can prime the body’s protective systems to be more responsive to the threat-related stimuli that can lead to pain [17,18,19,22,23].

Pain can be conceptualised as a response to a disturbance of homeostasis [24]. This disturbance is recognised through processing of available interoceptive and exteroceptive information. In simple terms, interoception is the awareness of sensations within the body. Therefore, interoception is implicated in any study of pain, as this involves the presence of threat or harm to the body [25].

Desmedt and colleagues provide a recent and comprehensive explanation of interoception as “the top-down and bottom-up processes by which an organism senses, interprets, and integrates signals from within itself and below the skin, across conscious and nonconscious levels” ([26] p. 87). The processing that leads to pain involves a distributed network of neurons across peripheral and central nervous systems, integrated with the immune and endocrine systems. Ascending neural pathways transmit signals from peripheral organs via the dorsal root ganglia and spinal cord or the vagal nerve to the brain [27]. Chen and colleagues also describe regulatory signals which connect the brain to the peripheral organs using both neural and non-neural descending pathways [27]. Thus, central modulation can amplify or attenuate bodily signals [27,28].

According to the biopsychosocial model of pain, pain is the product of the complex interplay of sensory experiences, beliefs, memories, and emotions and social context. This informs the allostatic interoceptive predictions that shape how a person interprets their pain and how they cope with it [17,19,25,29]. Therefore, when assessing the pain associated with breastfeeding it is important to be comprehensive and evaluate the broad range of contributing influences.

The Breastfeeding Pain Reasoning Model (BPRM) is a tool which reflects a biopsychosocial approach and addresses the broad scope of recognised pain mechanisms [30]. The BPRM assumes a shared contribution (and sometimes interaction) of the following domains in pain presentations:

The local stimulation referred to in the BPRM includes mechanical and chemical stimuli that trigger nociceptors. Nociceptors, receptors on free nerve endings, usually respond to high threshold stimuli that have potential to cause damage, although when sensitised they can react to lower-level stimulation. Mechanical forces-related tissue deformation (e.g., compression, traction, distension), tissue disruption, and changes in local endogenous chemical concentrations potentially lead to activation of nociceptors. Neuromodulating chemicals released in response to tissue damage as part of the inflammatory response can sensitise nociceptors, lowering the activation threshold. Importantly, nociceptor activation does not directly lead to pain but does provide the sensory information about danger to the body for the central processing of pain. That is, nociceptors are not pain receptors.

The external influences domain of the BPRM was adapted from the regional influences category of the original pain and movement reasoning model [31] to capture characteristics of the woman (e.g., nipple shape and flexibility) or child (e.g., tongue tie, small mouth) that might affect the interaction between mother and child and the mechanics of the interaction itself (e.g., latch). It also serves to capture other factors including issues due to the use of breast pump or pads and creams, and also the room temperature which may provoke vasospasm. This domain captures other factors that predispose, contribute to or exacerbate pathological processes and are not due to a pathological process.

The central modulation domain of the BPRM captures a broad array of factors that mediate or moderate the central processing of pain. Here, mediating factors change the relationship between the sensory information about danger by changing the arousal level of the body’s protection system (e.g., low mood, anxiety and fear, catastrophising, threatening environment). Pain and moderating factors can be considered pre-existing contexts (e.g., chronic poor health, history of traumatic events or adversity, epigenetic influences on phenotype) that suggest the body’s protection system may already be primed to respond, even before any sensory information about danger is received. This primed state has been described as a state of pain vulnerability, and it has similarities to the conceptualisation of allostatic load.

The aim of the BPRM is to capture the complex nature of pain by representing the relative contributions of each domain at a particular time as a single plot on the central gridded triangle [30]. This enables the clinician and the patient to visualise that the pain experience is multifactorial, and that each domain can enhance or reduce the experience. Moving beyond simplistic pathoanatomical reasoning, the BPRM reflects the dynamic, prediction-based processing and interpretation of interoceptive information in the context of real or perceived bodily threat [17,25,26,27].

We conducted this study to explore the perspectives of breastfeeding women regarding the causes and influences on lactation-associated pain. For the first time, we also aimed to determine if the theoretical domains of the BPRM align with women’s experiences of pain. A better understanding of pain during lactation may help women manage pain, prevent early cessation of breastfeeding, and optimise health outcomes for mother and child.

## 2. Materials and Methods

### 2.1. Research Design

This qualitative descriptive study used a phenomenological approach to explore individuals’ experiences and develop new meanings from their lived experiences [32]. Semi-structured individual interviews were performed. Data were analysed using a deductive approach, framed by the domains of the BPRM [30]. Additional themes and concepts were explored using inductive analysis.

In this article the terms women and breastfeeding are used, but we acknowledge that not all people who breastfeed identify as women.

### 2.2. Setting

This study was conducted in Singapore. A recruitment notice was placed on the social media page of the Breastfeeding Mothers’ Support Group, a charitable breastfeeding support organisation. The notice provided a study summary, screening tool, participant information sheet, consent form, and the research team e-mail contact. Eligible applicants who completed the online consent form were invited to participate.

This study was approved by the Singapore Institute of Technology’s Institutional Review Board (IRB number: 2022092). This study was performed in accordance with the Declaration of Helsinki.

### 2.3. Participants

Inclusion criteria were as follows: Singaporean citizens or permanent residents above 21 years old, able to speak and read English, ≥three weeks postpartum, had breastfed/expressed in the past three months, and experienced nipple/breast pain. The only exclusion criteria were women or children with major illnesses, such as major maternal depression or children with congenital heart disease. Convenience sampling was used. The early volunteers consisted mostly of highly educated women, working in health-related occupations and of Chinese ethnicity, so a second recruitment drive targeting underrepresented groups was attempted. The second notice was posted on the same social media page and asked for volunteers who did not have tertiary education, did not work in healthcare, and were non-Chinese ethnicity. Two additional participants from non-health related occupations were recruited.

### 2.4. Data Collection

An interview guide was developed based on the research question and the authors’ knowledge of the literature around pain and breastfeeding and refined during piloting. It included questions on demographic and pain characteristics, the 11-point verbal numerical pain rating scale [33], a flow of topics, and prompts related to the BPRM domains (Material S1).

Interviews were conducted between August and October 2022 via a secure videoconference platform and audio recorded. To preserve anonymity, participants were given pseudonyms. Two of the three trained female researchers (NSEC, SYL, VYTW) conducted each interview—one researcher led the interview while another supported the process. Post-interview, participants were provided details of relevant community support groups and a shopping voucher (SGD30) as compensation for their time.

### 2.5. Data Analysis

Audio recordings were captured and auto-transcribed using Zoom 5.10, with final transcripts checked for accuracy. Researchers ensured familiarity with transcripts and coded data independently following the thematic analysis process of Braun and Clarke [34,35]. One senior researcher, using Quirkos 2, a qualitative data management tool, coded all data. Other researchers, using NVivo R1.7, focused on individual BPRM domains. These analyses were combined in the final coding. Analyses were performed primarily using a deductive approach, framed by BPRM domains. Where participants’ experiences were unrelated to the BPRM framework, inductive analysis was performed, with additional codes created. Coding was crossed checked and synthesised into themes (Material S2, Coding tree). Verbatim quotes underwent minor modifications to improve readability.

### 2.6. Research Team

Members of the research team included experienced medically trained researchers (LHA, YPMN, ZA), lactation consultants (LHA, DF, YPMN), a pain expert (LEJ), and student researchers (SNEC, VYTW, SYL). The students, all females with no personal breastfeeding experience, undertook extensive training by experienced qualitative researchers (DF, LEJ) prior to study commencement. Researchers had no professional or social relationship with participants. LEJ and LHA are co-creators of the BPRM.

### 2.7. Quality of Study

Study design and reporting were performed according to the Consolidated Criteria for Reporting Qualitative Research (COREQ; Supporting Information: Material S3) [36]. Transcripts were checked for accuracy through listening to audio recordings, with additional checks during analysis to confirm unusual content. Analysis of data involved multidisciplinary collaboration to ensure codes and categories/themes were fully explored and interpreted [35]. Considering reflexivity, the team were aware of their differing understandings of pain concepts (LEJ as a pain expert) and experiences of providing breastfeeding support (LHA, DF, YPMN) in Singapore and Australia.

## 3. Results

### 3.1. Participants

Nineteen women were interviewed, with seventeen recruited in the first phase and two in the second phase. The interviews lasted an average of 46 min. One participant was found ineligible as her baby was 3 days old.

The mean age of participants was 32 years. Eleven of the eighteen participants were of Chinese ethnicity, consistent with the population of Singapore. Ten women reported on their first breastfeeding experience. The median age of the infants was 13 weeks. Most women were expressing breast milk as well as directly breastfeeding. Data saturation was achieved with eighteen participants. Table 1 presents participants’ characteristics.

### 3.2. Results of Analysis

**Figure 1:**
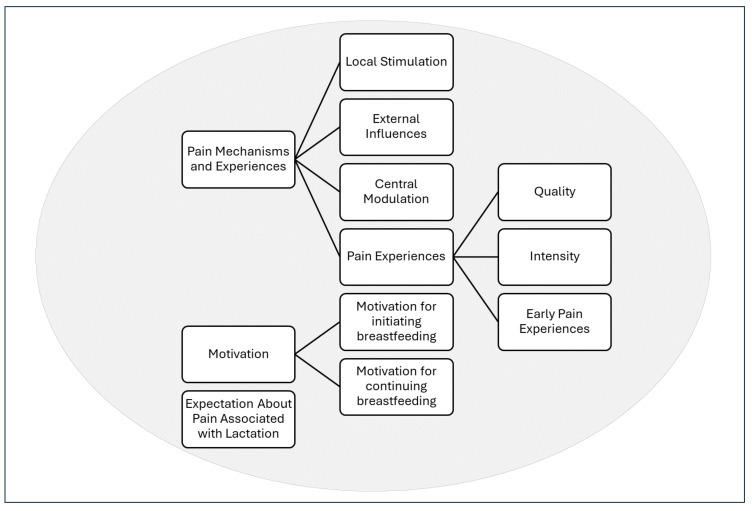
Themes and subthemes.

Deductive analysis identified evidence of alignment of women’s experiences with BPRM’s theoretical domains, which was further characterised by pain intensity and quality, with attribution by the participants to specific conditions or actions. These contributed to the main theme, “*Pain mechanisms and experiences*”; Figure 1 displays the subthemes and connected threads of this theme as well as the other two themes. Inductive analysis established two additional pain-related themes: “*Motivation to initiate breastfeeding and to continue despite pain*”, and “*Expectation about pain associated with lactation*”. Resilience was considered as a separate theme, but it showed interdependence with the other three themes. Table 2, Table 3 and Table 4 provide examples of quotes for each of the three themes.

### 3.3. Theme 1: Pain Mechanisms and Experiences

In this main theme, participants’ experiences were closely aligned with the BPRM domains of *local stimulation* and *external influences* (Table 2). Most participants did not readily relate their pain experience to factors in the *central modulation* domain even when prompted towards the end of the interview.

Women’s explanations that aligned with the *local stimulation* domain included abrasions and other signs of tissue injury, infection, and descriptions of increased internal biomechanical pressure on tissue, including swelling of the breast associated with onset of copious milk production.

Aligned with the *external influences* domain, women described nipple pain arising from friction from the infant’s poor latch, size mismatch between infant’s mouth and mother’s nipple, infant tongue-tie, and equipment issues. For equipment, pain was attributed to the type of equipment, pump settings (i.e., level of suction), duration of use, pump flange fitting difficulties, and incorrect nipple shield size.

It was women’s stories rather than their explanations of their pain that aligned with the *central modulation* domain. Their stories included events that potentially could enhance breastfeeding pain experience according to contemporary understanding of pain mechanisms. This included fever, traumatic birth experiences, lack of breastfeeding support, perceived injustice, fear of pain associated with breastfeeding, and low mood or anxiety. Some women agreed that tiredness, hunger, thirst, low mood, and stress could have worsened their pain experience by lowering their tolerance to pain. Many participants reported feeling overwhelmed and distressed by their painful experiences and some reflected on the challenges of parenting. While evident in their stories shared, the women did not readily think these events contributed to the pain experiences.

When sharing their understanding of pain mechanisms or causes, participants described their pain in quantitative and qualitative terms. Many reported extreme levels of pain intensity, and associated characteristics of the pain (sharp, shooting, burning, tingling, and aching) with underlying conditions (friction from infant’s poor latch, infection, engorgement, abrasions).

Some degree of pain was experienced by most women during early days of breastfeeding; nipple abrasions due to poor latching were often the cause of this pain. Some women believed that their nipples needed to become “used to” the infant feeding at the breast. Many women shared challenges associated with their early attempts at breastfeeding within the context of personal vulnerability. The pain experiences associated with early breastfeeding were reported as challenging, distressing, and for some women, even traumatising. Table 5 summarises women’s explanation of their pain as per the BPRM [30].

### 3.4. Theme 2: “I Have a Goal”—Motivation to Initiate Breastfeeding and to Continue Despite Pain

The other two themes were derived inductively. Theme 2 reflects notions of doing what is best for the baby and for self, fear of and sense of failure, and social pressures (Table 3). Participants described various motivations to breastfeed, mostly for the baby’s health and wellbeing. Other reasons were their perceived obligation as a mother, religious reasons, social expectations, and personal health benefits. Some participants understood the need to maintain breastfeeding or expressing to prevent pain from engorgement. Some described an emotional desire to bond with their child and avoid guilt. Some mothers mentioned that breastfeeding would be less costly than using commercial milk formula. These motivations helped mothers persist with breastfeeding, or expressing breast milk, demonstrating their resilience to reach their personal breastfeeding goals despite enduring extreme discomfort.

### 3.5. Theme 3: “Theory and Practical Is Really Different”—Expectation About Pain Associated with Lactation

Many participants had idealistic expectations of a pain-free natural process and reported feeling misled by information about breastfeeding received from nurses and lactation consultants, and from friends with breastfeeding experience (Table 4). Participants with previous breastfeeding experience also reported unexpected challenges; some explained this in terms of their newborn needing to learn to breastfeed.

Participants who experienced misalignment of their expectations with the reality of breastfeeding reported feeling emotional distress (injustice, distress, and low morale). These periods of vulnerability challenged their motivation to continue breastfeeding and led them to question their sources of information and support. Overall, the participants described that reframing their expectations, searching for multiple sources of information, and perseverance were the best strategies to overcoming breastfeeding challenges.

## 4. Discussion

This qualitative study explored the perspectives of breastfeeding women regarding the causes and influences of lactation-associated pain. The participants had clear, but not necessarily accurate, explanations for what caused their pain. Their explanations were aligned with the BPRM domains of *local stimulation* and *external influences*. *Central modulation* factors were evident in the women’s stories but only a few spontaneously articulated how *central modulation* factors influenced their pain. Pain and motivation, and expectations related to pain and discomfort, were apparent themes in the women’s stories. Resilience was another construct which was evident from participants’ stories which highlighted the interdependence between the themes of painful experiences, enhanced by strong motivation and by matching expectations with reality.

The descriptions and stories shared by the women in this study have similarities to those reported previously [37,38]. First-time mothers were surprised by the new sensations of breast fullness and nipple pain, as reported by Jackson et al. [10]. Their descriptions of pain using terms such as “shooting, burning, tingling, and aching” was also consistent with descriptions used by Jackson et al.’s participants [39]. While our interviewers did not probe to explore if the reported sensations were regarded as external or skin-level sensations or internal sensations from the breast itself, women did not seem to differentiate in terms of meaning of pain. This is an area for future research to understand interoceptive awareness of signals from within the internal lactating breast and to develop ways of measuring this subjectively and objectively. To date, the breast has not been discussed or defined as a peripheral organ capable of transmitting interoceptive sensory signals to the brain (e.g., see Figure 1 in [27].)

In this study, women reported battling with their motivation to continue breastfeeding, navigating issues of low milk supply, and the challenges of returning to work or study. Support, or lack thereof, from family, healthcare providers, or friends was also a common point reflected in the causal influences on breastfeeding duration from a study of Indonesian women [40]. A study that explored strategies for coping with breastfeeding pain reported resilience based on a perspective that pain would eventually end, motivation to tolerate pain for infant’s health, and dedication to reach breastfeeding goals [41].

This is the first time the BPRM has been used as a framework for analysis in research. Explanations of pain relating to biomedical or biomechanical processes were easily mapped to *local stimulation* and *external influences* domains. Explanations of pain reflecting *central modulation* were either elicited from participants or inferred from their stories. This suggests that the structure and content labels of the BPRM, reflecting a broad scope of pain mechanisms, can capture and explain lactation-associated pain.

Importantly, using the BPRM identified where women’s explanations did not reflect the complexity of pain. That is, participants did not readily link their pain experience with their negative cognitive, emotional, or social states such as psychological stress, inadequate sleep and fatigue, low mood, anxiety, injustice, and inadequate social support. This can be expected because firstly, due to the complex nature of pain, a direct cause is not always apparent [17,19]. Secondly, presence of a single factor, even obvious nipple tissue injury, is not always enough to explain pain [17]. Finally, it is hard to link pain to all domains without a biopsychosocial conceptualisation of pain. This challenges the predominant biomedical or biomechanical paradigms that many healthcare providers perpetuate in their interactions. Thinking of pain in this simplistic way, for example, focusing solely on localised tissue damage, limits the management options available. 

From an interoceptive perspective, bodily states reported by participants, such as tiredness, hunger, thirst, low mood, and stress, increase the burden of ascending internal signals. In situations of homeostatic strain, central regulatory systems (descending interoceptive/efferent pathways) may be less efficient, and attentional/salience networks may amplify internal distress signals. A possible result is that additional nociceptive input may be less tolerated, in part because the system is already taxed by ongoing internal demands, activating allostatic processes and priming pain responses [17,28]. Stress-management, focus on sleep, enhancing social and emotional support, and targeted education and reassurance should be seen as pain management strategies along with the common physical tissue-focused approaches.

Our findings highlight the need for healthcare providers supporting breastfeeding women to enact biopsychosocial ways of thinking, educating, and managing lactation-associated pain [30]. This is already happening in other pain contexts [42] and includes processes and strategies that optimise support, reduce fear, and promote resilience.

Educators of healthcare professionals should ensure that biopsychosocial models of pain are taught, and that new concepts in pain and interception are incorporated into teaching. In particular, clinicians and peer supporters providing lactation care must become familiar with these concepts so they can provide holistic care for women experiencing pain with breastfeeding. Education should extend to family members, support persons, employers, and other influential individuals [8].

Limitations of the study included a recruitment strategy that only targeted a single online support group, which may have contributed to lack of diversity. Participants all had a tertiary qualification; many were from health or related fields and a single ethnic group. Our attempt to rectify this with a targeted recruitment had limited success, and time limitations due to the unfunded nature of the project prevented further pursuit of this. The interviews provided an opportunity to share stories of injustice; some women hoped that sharing their stories might bring positive change. This may affect how stories were told and events portrayed. Some women were reporting experiences during a time of staff and service restriction during the COVID-19 pandemic when negative experiences of service have been reported, including lack of support [43,44].

There is an opportunity for research in educating women and their support network about the physiological changes associated with the onset of lactation and breastfeeding, as well as the multidimensional nature of breastfeeding pain; of note, higher-order interpretation of perceived bodily signals (i.e., interoceptive appraisal and attribution) is underexplored in this relationship but likely plays a fundamental role [45]. Using an interoceptive lens could help to illustrate how signals from the breast inform the brain, and how social, emotional, and cognitive factors influence the individuals’ perception of painful experiences [25,27]. The response to pain is also individual and contextual [23]. The conscious awareness and the attribution and appraisal of pain and associated feelings can be modified by contexts that create anxiety or uncertainty [22,29,45]. These contexts featured in participants’ stories related to mismatch of expectations and feeling unsupported. By considering the broad influences on pain, healthcare providers can develop policy and practice guidelines that prioritise strategies to address the psychological and social challenges breastfeeding women experience, and thus improve health outcomes for the breastfeeding dyad and mother–child interactions.

## 5. Conclusions

Our study highlights the complex nature of pain associated with breastfeeding. Pain, which can be extreme, was mostly attributed to biomechanical or biomedical mechanisms, downplaying psychological and social influences. Healthcare providers supporting people who are breastfeeding need to understand the multidimensional nature of pain and use interventions that set realistic expectations, optimise support, reduce fear, and promote resilience. Enacting a biopsychosocial approach to pain may improve management of breastfeeding pain and breastfeeding outcomes for breastfeeding dyads.

## Figures and Tables

**Table 1:** Characteristics of participants (*n* = 18).

Characteristics	
Age in years, mean ± SD (range)	32 ± 4 (22–42)
Ethnicity, *n* (%)	
Chinese	11 (61.1)
Malay	4 (22.2)
Indian	1 (5.6)
Unspecified	2 (11.1)
Educational level, *n* (%)	
Tertiary diploma or higher	18 (100)
Support at home, *n* (%)	
Spouse only	1 (5.6)
Spouse and family ^a^	6 (33.3)
Spouse and helper ^b^	7 (38.9)
Spouse, family, and helper	4 (22.2)
First time breastfeeding, *n* (%)	10 (55.6)
Child’s age in weeks, mean ± SD (median; range)	27.7 ± 28.3 (13; 4–112)

^a^ Parents, parent in-laws or extended family (including regular role in care at home). ^b^ Helpers, confinement nannies or any external help (including regular part-time support at home).

**Table 2:** Theme 1 Pain mechanisms and experiences.

Subthemes	Supporting Quotes
Local Stimulation	So, there shouldn’t be any friction or abrasion. I think that’s what’s causing the pain there and all the wounds. (Eleanor) The pain is because more milk is trying to come in— they’re already filled to capacity; that kind of the stretching and all that. (Felicity)
External Influences	If your position is awkward and the baby is uncomfortable, the baby will tend to unlatch, and you would get a shallow latch which would lead to the pain. (Blake) The lactation lady also said, ‘You know her mouth was still very small so it’s not fully, like you know, enclosing the nipple’. (Holly) When you pump it’s like torture—every [pish sound], every suction, it’s very painful. They say you have to select the correct flange size and all those things. (Tina)
Central Modulation	If I haven’t eaten or drunk … or not enough sleep … I’ve not [been] able to latch the baby properly or even uh, when I’m busy with other things … then once the baby latch on, then the pain comes then I felt that pain [is] more intense than normal, yeah. (Judith) Yeah, I think it was a couple of occasions, but I didn’t think it was related. But now that it happens to me more … I think there’s definitely a correlation between my stress and more pain, yeah. (Holly) It gives a lot of tension because you want to tend to the child immediately … So, you’re just panicking in a way, so that can affect the overall wellbeing of the breastfeeding journey, which could affect the pain in that sense. (Mandy)
Pain experiences: quality	Yeah, that’s the shooting pain for the clogged duct. (Clarissa) You can feel like the needle poking kind—so mostly on the one that is very badly engorged. (Lisa)
Pain experiences: intensity	It’s so bad until you cannot wear a bra over it ... I mean loose clothes still can. But you cannot have any pressure on the boobs. (Tina) I think for the sharp pain, it would be a 7 to 8 (out of 10). However, that moment is very brief. So the moment the baby starts sucking, then the pain will fade off. (Blake) So, the initial latch on, it’s like 8, 9/10—so it’s like the toe-curling pain that you, you are just waiting for it to pass [laughter] and after that, then it ease off then you can sense that baby is drinking the milk. (Judith)
Pain experiences: early experience	I also know that it is normal for a newborn, at the newborn stage, it can be painful because it is still sensitive and not seasoned yet. (Mandy) It’s my third time already, so I kind of know what breastfeeding is like. But for the baby, he is still new to it. (Eleanor)

**Table 3:** Theme 2 Motivation to initiate breastfeeding and to continue despite pain.

Subthemes	Supporting Quotes
Motivation for initiating breastfeeding	Because all the books I read when I was pregnant, about taking care of baby, all recommend breastfeeding until the baby turns one. And most my friends also do breastfeeding. (Tina) As a dietician … we encouraged our pregnant mummies to breastfeed then you ‘jiang jui (say very long)’, you are self psycho-ed also. Also, of course, to save milk powder, it does save a lot of costs. (Orphelia)
Motivation for continuing breastfeeding	Okay, because for us for Muslims, we believe that we should feed our child until the age of two. So that’s my ultimate goal, but in the end, even before it reaches two, I got pregnant (laughs). (Clarissa) Six months, according to WHO, it’s like recommended to exclusively breastfeed for six months at least—before introducing any formula or what, … for my first child, I didn’t breastfeed for very long, and I find that she fall sick more often and the severity of the illness was quite bad—she has asthma and eczema. (Judith) First, if I don’t feed her or I don’t pump, the pain will get worse. So if you don’t do it, you will feel more miserable. And so, then you will be like, ‘okay let’s just do it’. And then the second thing is because you know is very strange … after delivery it affects the way you’re think about what you want, for your child. So that guilt feeling and that closeness that you want to have for your child are the main motivating factors. (Mandy) Actually, the nurse was seeing that I was in great pain and they keep offering formula top up … I tell myself if I give in once right, then second time, I will keep giving in. (Orphelia) So, my husband was telling me, then you just give her formula—actually I really wanted to—but I told him like, but I have a goal—like a year of breastfeeding my child. I was very determined to reach that goal. (Nina)

**Table 4:** Theme 3 Expectation about pain associated with lactation.

Subthemes	Supporting Quotes
Expectations	I have a friend a young mom … she has three kids already—so every time I see her, she will just whip out only, then she like, “Ya, very easy! Baby want milk you just have to feed her, don’t need to make milk.” Then when I had baby, I was like “Wah, I cannot do that!” (Stella) I think there’s an overhype of how good breastfeeding is. Then—there it’s just not enough to teach mothers the reality of how difficult, tiring and painful breastfeeding is … (Mandy) I am a trained counsellor with the BMSG (Breastfeeding Mother’s Support Group). I know all the breastfeeding knowledge and I also help with the new moms there. But theory and practical is really different. While I know all the theory and I also know what is normal at the newborn stage, it can be painful because it is still sensitive and not seasoned yet. But when I’m actually doing it, it is a very different experience. (Eleanor)

**Table 5:** Factors contributing to pain as reported by participants and aligned with BPRM ^1^ domains.

Domain	Factors Influencing Pain	Inferred Contributors ^2^
Local Stimulation	Abrasions/nipple damage Bruising of the nipple Infection Breast engorgement Localised breast inflammation Mastitis “Refilling pains”	
External Influences	Poor latch or biting Friction Exposure to cold Infant tongue-tie Baby with small mouth or unable to open wide Baby learning to latch Emotionally upset child Size of flange of breast pump Size of nipple shield Shape/size of nipple Ill-fitting bra Clothing on sensitive area of breast/nipple Lack of access to oils and balms Massage (self and other) to engorged breasts	
Central Modulation	Lack of understanding from husband Not used to pain Anticipation of painful experience Focus on painful experience Not feeling well Frustration or panic Stress Lack of sleep and tiredness	Frustration Injustice Helplessness Fear Constant pain Fever

^1^ BPRM: Breastfeeding Pain Reasoning Model [30]. ^2^ Inferred contributors are those which were evident in participants’ stories but not mentioned by participants as relevant to their pain experience.

## Data Availability

Transcriptions of the original, anonymized transcripts can be obtained from the lead author, subject to a reasonable request, due to restriction by the ethics committee.

## Supplementary Materials

The following supporting information can be downloaded at: https://www.mdpi.com/article/10.3390/brainsci15101087/s1, Material S1: Interview Guide (word document), a semi-structured interview guide that was used for the individual interviews of the participants; Material S2: Coding tree (Word document), coding tree for the three themes: pain mechanisms and experiences, motivation and expectations; Material S3: COREQ (Word document), consolidated criteria for reporting qualitative studies (COREQ) checklist.
