# Supplementary material for: Do Lactating Mothers’ Descriptions of Breastfeeding Pain Align with a Biopsychosocial Pain Reasoning Tool? A Qualitative Study
Source: Brain Sci. 2025 Oct 8;15(10):1087. doi: 10.3390/brainsci15101087 (PMC12563564; doi:10.3390/brainsci15101087)
Supplement: Supplementary file 1 [file brainsci-15-01087-s001.zip › brainsci-3848813-supplementary.pdf]

Supplementary Material S1: Interview Guide

1. Friendly welcome and housekeeping

1	Welcome	Hello!! Thank you for your interest in participating in our study, I am _______ (introduce ourselves normally).
2	Introduction of researchers	
3	Explain aims of project	We are conducting this project to learn more about women's experiences of pain while breastfeeding or expressing.
4	Reinforce how their contribution will help	We are interested in your thoughts and so we will be asking you some questions. There are no right or wrong answers, and your experiences will be added to the experiences of the other women we are interviewing.
5	Reassure them of confidentiality	I would also like to reassure you that everything you say to us will be kept confidential and used strictly for research purposes. All the information we collect during this interview will be de-identified, so your personal information will be kept secure.
6	Check that they entered with their assigned pseudonyms	[if not done]<br>Before we go on, we will re-name you with a pseudonym.
7	Reminder about recording	I'd like to remind you that we will be recording only the audio from this interview, is this still alright with you?
8	Permission to begin	Now I've explained the aims and details of this interview, are there any other questions?<br>Is this a good time for you?<br>Are you ready to begin now?<br>Just let us know if you need to stop along the way.

2. Questions

Topic	Prompts	Further prompts
**Background Questions**<br>As you know, we are interested in hearing about women's experience of breastfeeding or expressing breastmilk and will be asking you some questions to better understand your perspective.		
1. Demographic questions	See sheet 6a	
2. Overall breastfeeding experience	Can you tell me about your recent experience of breastfeeding, starting when the baby was born?	Can you describe the first feed?<br>How were the first days of breastfeeding?<br>And how did it go when you first got home from hospital?<br>And then what happened?

		How are things going now?<br>(<u>not</u> necessary if already indicated she has stopped)
2. Follow-up (if not elicited in story)	• breastfeeding and/or expressing?<br>• how frequent?<br>• if stopped, when?<br>• have mother and the baby been well?	

**Topic**	**Prompts**	**Further prompts**
**Pain-related questions**<br>Now we will be moving on to ask you some questions regarding the pain you have experienced.		
3. Pain<br><br>3. Pain … cont.	You mentioned that you had pain. Can you tell me more about this?	Can you describe the pain associated with breastfeeding?<br>Where did you feel pain?<br>When does the pain(s) come on?<br>How long does/did the pain last?<br>How would you describe the intensity of the pain?<br>How long have you been getting this/these pains?<br>How would you describe the quality or type of pain(s) you feel/felt?<br>[If reply not forthcoming offer 'burning or sharp or aching']
	What do you think is the cause of the pain(s) you are feeling?	What do you feel was going on in the [nipple or breast] itself?<br>Are there other things that make your pain worse?<br>Anything else?<br>Some women have found the position they feed their baby in or the baby's position affects their pain – have you found similar things affect your pain?<br>Some women have found their mood, or how tired they are, affects their pain – are there similar things that you have noticed affects your pain?<br>Some women have found the level of support they have from others can affect their pain – is this something you can relate to with your pain?

4. Pain management	Was there anything that helped reduce the pain?	Are there other things that helped? What else did you try?<br>Did you seek help? Who did you seek help from?<br>What advice have you received that has helped you with your pain?
5. Breastfeeding expectations	Prior to breastfeeding this child, what did you expect?	What influenced you and your expectations about breastfeeding? Duration, direct or expressing, timing, volume, etc.

3. Wrapping up

1	Closing	Okay, we have come to the end of our interview.<br>Thank you very much.
2	Check if there's anything else they would like to share	Is there anything else you would like to add?<br><br>If not, do you have any questions for us?
3	Indicate recontact for clarification of transcript	Can we just check with you that it would be ok for us to recontact you, if we need to clarify anything that you have shared today? Would that be ok?
4	Acknowledgement & Thanks	Thank you for your time.<br>We really appreciate your willingness to share with us.<br>This has been a great help to our research project.
5	Notify that you will pass on relevant details of local breastfeeding support	We will send through the details of some local breastfeeding support groups and also an e-voucher to compensate you for your time.<br>Thanks again for all that valuable information, that will be all, goodbye.

Supplementary Material S2: Coding Tree (Pain and pain-related themes)

Themes	Category	Sub-category	Codes
Pain mechanisms and experiences	Pain	Pain mechanisms	Local stimulation<br>External influences<br>Central modulation<br>Inferred cause<br>Pain experiences (*quality/intensity*)
Motivation to initiate breastfeeding and to continue despite pain	Motivation		Motivation (to start/*to persist*)<br>Draining life energy/overwhelming<br>Feelings of failure<br>Ending breastfeeding—impact
Expectations about pain associated with lactation	Expectations versus reality	Expectations – realistic and idealistic	Early breastfeeding experiences<br>Prior breastfeeding experiences<br>Current breastfeeding experiences

Supplementary Material S3: Consolidated criteria for reporting qualitative studies (COREQ) Checklist

Consolidated criteria for reporting qualitative studies (COREQ): 32-item checklist

Adapted from: Tong A, Sainsbury P, Craig J. Consolidated criteria for reporting qualitative research (COREQ): a 32-item checklist for interviews and focus groups. *International Journal for Quality in Health Care*. 2007. Volume 19, Number 6: pp. 349 – 357

**No. Item**	**Guide questions/description**	**Reported on Page #**
**Domain 1: Research team and reflexivity**		
Personal Characteristics		
1. Interviewer/facilitator	Which author/s conducted the interview or focus group?<br>Nicole Shi En Chew (NSEC)<br>Shi Yun Low (SYL)<br>Victoria Yu Ting Woo (VYTW)<br>Lester E. Jones (LEJ) (training/supervision)<br>Doris Fok (training)	5
2. Credentials	Three interviewers were Honours students undertaking their research training<br>PI (LEJ)—researcher with experience in focus group and interview<br>Doris Fok—researcher with experience in focus group and interview; breastfeeding consultant	5
3. Occupation	What was their occupation at the time of the study?<br>Honours students in training:<br>Nicole Shi En Chew<br>Shi Yun Low<br>Victoria Yu Ting Woo<br>Lester E Jones—Senior Lecturer, Health and Social Sciences Cluster<br>Doris Fok—Lactation consultant/Fellow in department of Obstetrics and Gynaecology	Title page

	Lisa Amir—Professor, School of Nursing and Midwifery<br><br>Yvonne Peng Mei Ng—Senior Consultant in Department of Neonatology<br><br>Zubair Amin—Associate Professor, Department of Paediatrics	
4. Gender	Was the researcher male or female?<br><br>Female interviewer.	5
5. Experience and training	What experience or training did the researcher have?<br><br>Student researchers undertook supervised training, overseen by LEJ, Doris Fook, including volunteer participants known to the research team.	5
Relationship with participants		
6. Relationship established	Was a relationship established prior to study commencement?<br><br>No participants were known to the research team.	5
7. Participant knowledge of the interviewer	What did the participants know about the researcher? e.g. personal goals, reasons for doing the research.<br><br>Participants were made aware of the following: “The purpose of this Research is to explore the perspectives of women regarding the pain experienced with lactation. The findings gathered from this study will provide an in-depth view of Singaporean women’s pain experiences and will inform future research examining better ways to support breastfeeding women”	Notes from Participant Information Sheet<br><br>Not reported in manuscript
8. Interviewer characteristics	What characteristics were reported about the interviewer/facilitator? e.g. bias, assumptions, reasons and interests in the research topic.<br><br>PI (LEJ) and Professor Amir are co-creators of the Breastfeeding Pain Reasoning Model	5
**Domain 2: study design**		
Theoretical framework		
9. Methodological	What methodological orientation was stated	

orientation and theory	to underpin the study? e.g. grounded theory, discourse analysis, ethnography, phenomenology, content analysis.<br><br>Phenomenological, deductive, and inductive thematic analysis.	4 & 5
Participant selection		
10. Sampling	How were participants selected? e.g. purposive, convenience, consecutive, snowball.<br><br>Initial phase—convenience sampling, Second phase—purposive sampling.	4
11. Method of approach	How were participants approached? e.g. face-to-face, telephone, mail, email.<br><br>Advertisement on social media of local breastfeeding support group.	4
12. Sample size	How many participants were in the study?<br><br>Total of18 participants.	7
13. Non-participation	How many people refused to participate or dropped out? Reasons?<br><br>One interested person could not be included as age of infant did not meet inclusion criteria.	6
Setting		
14. Setting of data collection	Where was the data collected? e.g. home, clinic, workplace.<br><br>Recorded audio files via institutional licensed Zoom account.	5
15. Presence of non-participants	Was anyone else present besides the participants and researchers?<br><br>Two researchers were present—one who led the interview and the other who	

	monitored technology and the responses, including ensuring intended questions were asked.	5
16. Description of sample	What are the important characteristics of the sample? e.g. demographic data, date.<br><br>11/18 Chinese ethnicity<br><br>18/18 Tertiary diploma or degree<br><br>10/18 First breastfeeding experience<br><br>13 weeks infant median age	6<br><br>Table 1
Data collection		
17. Interview guide	Were questions, prompts, guides provided by the authors? Was it pilot tested?<br><br>Interview guide was developed and modified during interview training and pilot tested with three women with recent breastfeeding experience.	Appendix B – Interview Guide<br><br>6
18. Repeat interviews	Were repeat interviews carried out? If yes, how many?<br><br>No.	-
19. Audio/visual recording	Did the research use audio or visual recording to collect the data?<br><br>Online video interviews were performed but only audio was collected.	5
20. Field notes	Were field notes made during and/or after the interview or focus group?<br><br>Notes on any significant observations and process were shared verbally with PI after each interview.	Not reported in manuscript
21. Duration	What was the duration of the interviews or focus group?<br><br>Average duration of each interview was 46 minutes.	6

22. Data saturation	Was data saturation discussed?<br><br>After the first phase of interviews, it was felt that there were potential gaps in racial diversity and education level. A second round of interviews took place but with limited response/adjustment to sample. Despite this, during the coding process, the number of new codes required reduced over time and data saturation was reached with 18 participants.	6
23. Transcripts returned	Were transcripts returned to participants for comment and/or correction?<br><br>Transcripts were not returned to participants for comment or correction.	Not reported in manuscript
**Domain 3: analysis and findings**		
Data analysis		
24. Number of data coders	How many data coders coded the data?<br><br>Data was coded initially by Honours students (one student per domain) and the PI, independently, then reviewed by the external members of the research team.	5
25. Description of the coding tree	Did authors provide a description of the coding tree?<br><br>Yes.	Appendix D<br><br>– Coding Tree
26. Derivation of themes	Were themes identified in advance or derived from the data?<br><br>Themes were derived from data but Theme 1 reflects deductive process based on BPRM.	5
27. Software	What software, if applicable, was used to manage the data?<br><br>Institutional licensed software:<br>Quirkos and NVivo for data management.	5
28. Participant checking	Did participants provide feedback on the findings?<br><br>No.	-

Reporting		
29. Quotations presented	Were participant quotations presented to illustrate the themes/findings? Was each quotation identified? e.g. participant number.<br><br>Yes, and identified with pseudonym.	Tables 2,3,4
30. Data and findings consistent	Was there consistency between the data presented and the findings?<br><br>Yes.	Tables 2,3 4
31. Clarity of major themes	Were major themes clearly presented in the findings?<br><br>Yes—Theme 1.	9<br><br>Table 2
32. Clarity of minor themes	Is there a description of diverse cases or discussion of minor themes?<br><br>Yes—Themes 2 and 3.	11, 12<br><br>Tables 3,4
